# Progress in Genito-Urinary Surgery

**Published:** 1903-10-31

**Authors:** 


### Progress in Genito-Urinary Surgery.

**Ureteritis in the Female.**—Garceau[1] has contributed a valuable paper on this subject. Acute ureteritis is usually a part of a pyelitis or cystitis, and cannot be detected. Diphtheritic ureteritis has been found at autopsies. Chronic ureteritis may be the sequel of either a vesical or a renal inflammation. It would be more common if the valve at the vesico-ureteral opening did not act as a determined barrier to the entrance of germs. As an illustration of this protective power the author cites a case in which after nephrectomy of one side the bladder remained infected with tubercle for five years before the second kidney became attacked. That fluids injected slowly into the bladder occasionally pass into the ureters has been proved by experiment. The gonococcus is the germ most likely to cause ureteritis on account of its tendency to cause stricture. If the kidney is infected in a case of ureteritis renal pain may be expected; but as the bladder is nearly always affected the symptoms are mainly referred to that organ, and the commonest symptom is frequency of micturition. The desire to micturate never leaves the patient, and it constitutes the principal source of suffering. There may be deep pain in the bladder with micturition on the side corresponding to the affected ureter. An excessive degree of neurasthenia is frequently developed, caused by the incessant suffering and loss of sleep. The most important sign is the tenderness on pressure of the affected ureter from the vagina. With this there is coincident extreme desire to urinate. Also, in long-standing cases, some enlargement of the ureter may be discovered vaginally. Cystoscopy will usually reveal, with or without patchy cystitis and swelling of the trigone, a swelling of the ureteral eminence on the affected side, with redness, swelling, and puffiness of the opening of the ureter. The discovery of excess of desquamated epithelium in the urine, which may or may not be associated with pus, is considered to be of great diagnostic importance. The Kelly cystoscope with oblique end is considered to be the only serviceable instrument for collecting the urine from the ureters free from admixture with vesical elements and from elements due to trauma. The excretion of urea is usually diminished on the affected side. Simple ureteritis may be confounded with ureteritis together with stricture of the ureter. Passing bougies will settle the question as to the existence of stricture. Most frequently ureteritis is not recognised at all, and the cases are treated as examples of chronic cystitis or as reflex bladder troubles of nervous origin. The prognosis is bad unless the affection is detected early and properly treated. In treatment any co-existing disease of the pelvic organs must be cured. General attention to diet and hygiene is important. Santal oil, urotropine, and bicarbonate of soda in large doses may be useful. Trional is good for sleeplessness. Topical applications to the bladder have done much good in these cases. Nitrate of silver solution in a 5 or 10 per cent. strength applied to the trigone and ureteral eminence is useful, and also bladder irrigation with 5 per cent. protargol or 50 per cent. ichthyol solution. The rational treatment is by means of topical applications in the ureter. The solutions are injected through a straight silver tube connected with a bottle containing the fluid by a rubber tube. By means of a rubber handbag, also connected with the bottle, the fluid is driven through the silver tube by air pressure. A 2 per cent. boracic or 1 per cent. silver nitrate solution may be used.

**Bilharziosis of the Genito-Urinary Organs.**—As cases of this disease in people who have been abroad are met with from time to time in this country, *e.g.* in some of the soldiers who went through the Boer War, we draw attention to a paper on the subject by F. Milton of Cairo.[2] The writer points out that bilharziosis develops in two distinct ways, though these may be combined in the same case. In one form—the atrophic—there is destruction of the mucous membrane and glandular tissue of the affected part, with the formation of fibrous tissue. In the other form—the hypertrophic—there is great overgrowth of the normal elements of the part attacked, with the formation of polypi and outgrowths, a tissue being produced which might be called bilharzial tissue. The symptoms of the disease in the bladder are hæmaturia at the end of micturition, with pain and scalding. These are early symptoms, and in most cases remain the only symptoms. In others intractable cystitis develops and loss of function of the bladder with constant tenesmus. The development of a calculus is common. Adhesions of the bladder to the anterior abdominal wall and fistulæ may develop. The ureters may be obstructed. Death occurs from exhaustion, or disorganisation of the kidneys, or septicæmia. The stage of hæmaturia is best treated with the liquid extract of male fern given internally. Cystitis may be treated by washing out the bladder with antiseptics, but astringents have little effect. Also salol, urotropin, or methylene blue may be given. Calculi should be removed by lithotrity, not by a cutting operation. Perineal drainage of the bladder may have to be resorted to. The usual evidence of bilharziosis of the urethra is the formation of a fistula either on the roof, which is commoner, or on the floor of the urethra. Both forms lead to openings in the perineum, but the symptoms and septic complications are more marked in the "floor" fistulæ. The treatment consists in free excision of the fistulæ. The author further discusses bilharziosis of the rectum and of the female genital organs.

**Senile Enlargement of the Prostate.**—Several papers on this subject have recently appeared in British journals. Sir Wm. Thompson,[3] reviewing the history of supra-pubic prostatectomy, points out that of 24 cases reported by McGill and his colleagues at Leeds in 1889 only about seven did not require a catheter afterwards. These cases also, in the main, were partial removals by scissors and forceps. But in this country the operation had practically been abandoned until P. J. Freyer recently reported his highly successful cases in which the gland was completely removed. Five cases are reported by Thompson. Four were successful. In one of the latter the entire prostate and prostatic urethra were removed. Discussing the perineal mode of operating, it is pointed out how difficult it is to reach the upper limit of a moderately enlarged prostate. It has been said that an essential possession of the surgeon for this operation is that of a forefinger with a working length of three inches at least. But even then only two-thirds of the cases could be dealt with through the perineum. Another objection is that the bladder cannot be completely explored with the finger passed through the perineum, and calculi may thus be missed. Thompson further draws attention to the following points: (1) The lateral lobes are most frequently the cause of obstruction. (2) The bulk of the prostate, as felt in the rectum, gives no indication of its intravesical contour. (3) The size of the prostate has no necessary relation to the severity of the urinary distress. (4) The smaller the tumour the more difficult, relatively, is its enucleation. The larger tumours seem to be more loosely attached. P. J. Freyer[4] has now published a sixth series of cases of total extirpation of the prostate, bringing the total number of cases operated upon up to 45. Of these 40 have been completely successful. There were five deaths, viz., two from mania, one from heat-stroke, one from uræmic coma, and one from pneumonia. F. A. Southam,[5] states that cystotomy and bladder drainage is a valuable means of affording temporary relief in prostatic cases where there is much cystitis and the introduction of a catheter is painful and difficult. He much prefers to treat the cases of sudden retention of urine in this way rather than by aspiration. Also drainage may be adopted as a permanent measure where the patient is too old or too weak to bear prostatectomy. A permanent supra-pubic drain may be applied with Thompson's silver shield or with a vulcanite tube through which a soft rubber catheter passes into the bladder, the other end being connected to an indiarubber urinal attached to the patient's leg. In one of the author's cases the patient wore such a tube for six years with complete relief to his symptoms and without inconvenience. The author has performed castration in a number of cases, but, except in a few, improvement was slight or absent. A case of total extirpation of the prostate by the author is mentioned in which the gland was fibro-myomatous, not adenomatous, and the enucleation was difficult. The gland came away finally in its capsule, together with the prostatic urethra. There was severe shock, suppression of urine, and death on the third day. The author states that in a future case of the kind he would leave the prostate alone and drain supra-pubically. He considers Freyer's operation superior to "intracapsular enucleation," in which the separate adenomatous growths are shelled out of the rest of the gland. Bruce Clarke,[6] in a paper on "prostatic obstruction," advocates the use of the above term rather than that of "prostatic enlargement," because in many cases the patient finds it quite impossible to empty the bladder, though there is no enlargement of the prostate. In some of these cases the prostates are hard, and may or may not be enlarged, and the author regards them as dependent upon quite a different pathological process to that producing the enlargement of the gland with adenomata. The hardened prostatic tissue may be compared to the hardened mammary-gland tissue in chronic mastitis. A third cause of prostatic obstruction, the author points out, is a development of enlarged veins in the prostate, on the trigone, and around the vesical orifice. A fourth cause is inflammation and congestion of the mucous membrane surrounding the urethral orifice. The treatment recommended for these hard prostates is that of removing one half of the gland. The author appears to think it likely that in the future catheter life will cease to exist because early operative treatment will come into vogue. W. P. Purvis[7] records a case in which he removed a large portion of the prostate, weighing six and a half ounces, by the supra-pubic method, for retention of urine. The mass was removed in four pieces, and a good recovery finally ensued, though certain complications arose in the first few weeks.

**Cancer of the Prostate.**—Reginald Harrison[8] believes that carcinoma of the prostate is far more common than it is often considered to be. The recognition of the condition in the early stages is difficult. Not infrequently it is met with in persons at a younger age than those in whom the ordinary forms of enlargement of the prostate are met with. It is usually associated with lumbar and sciatic pain. The glands in the groin tend to be affected. Rectal examination shows the gland to be of stony hardness and very fixed. Harrison has had several cases in which the local symptoms were slight and the course of the disease slow, and death resulted from general decay rather than from the interference with the functions of the bladder. Carcinoma of the prostate resembles scirrhus of the breast in its hardness. The cystoscope is considered to afford valuable help in differentiating cancerous prostatic disease from the other forms of enlargement. The author then relates two cases of carcinoma in which he attempted to remove the gland. In each there was rapid recurrence. High-frequency currents and Roentgen rays are recommended as methods of treatment. Partial operations on the prostate are recommended for those forms of non-cancerous disease in which one particular part of the gland is hypertrophied and is the cause of all the symptoms.

[1] Amer. Jour. Med. Science, Feb. 1903, p. 285. [2] Lancet, March 28, 1903, p. 816. [3] Brit. Med. Jour., April 18, 1903, p. 895. [4] Ibid., July 4, 1903, p. 3. [5] Ibid., April 18, 1903, p. 901. [6] Ibid., July 4, 1903, p. 7. [7] Lancet, April 25, 1903, p. 1162. [8] Brit. Med. Jour., July 4, 1903, p. 1.

